# Ethnic diversity, poverty and social trust in Germany: Evidence from a behavioral measure of trust

**DOI:** 10.1371/journal.pone.0199834

**Published:** 2018-07-18

**Authors:** Johanna Gereke, Max Schaub, Delia Baldassarri

**Affiliations:** 1 Carlo F. Dondena Centre for Research on Social Dynamics & Public Policy, Bocconi University, Milan, Italy; 2 Wissenschaftszentrum Berlin für Sozialforschung (WZB Berlin Social Science Center), Berlin, Germany; 3 Department of Sociology, New York University, New York, United States of America; Leibniz Institute for Prevention Research and Epidemiology BIPS, GERMANY

## Abstract

Several scholars have concluded that ethnic diversity has negative consequences for social trust. However, recent research has called into question whether ethnic diversity per se has detrimental effects, or whether lower levels of trust in diverse communities simply reflect a higher concentration of less trusting groups, such as poor people, minorities, or immigrants. Drawing upon a nationally representative sample of the German population (GSOEP), we make two contributions to this debate. First, we examine how ethnic diversity at the neighborhood level–specifically the proportion of immigrants in the neighborhood–is linked to social trust focusing on the compositional effect of poverty. Second, in contrast to the majority of current research on ethnic diversity, we use a behavioral measure of trust in combination with fine-grained (zip-code level) contextual measures of ethnic composition and poverty. Furthermore, we are also able to compare the behavioral measure to a standard attitudinal trust question. We find that household poverty partially accounts for lower levels of trust, and that after controlling for income, German and non-German respondents are equally trusting. However, being surrounded by neighbors with immigrant background is also associated with lower levels of social trust.

## Introduction

Over the last decade, scholars in political science, economics, and sociology have repeatedly debated whether ethnic diversity constitutes a threat to social cohesion. While a few empirical studies have concluded that ethnic diversity has a negative effect on trust in the U.S. [1–2], as well as in some European countries [3–8], this line of research has been challenged on both conceptual and methodological grounds [9–13]. This paper makes two contributions to this debate. First we examine how ethnic diversity at the neighborhood level–specifically the proportion of immigrants in the neighborhood–is linked to social trust focusing on the compositional effect of poverty. Second, we use a novel behavioral measure of trust from a trust game embedded in a large-scale survey with a representative sample of the German population together with newly available fine-grained contextual data on neighborhood ethnic diversity and poverty.

Much of the empirical literature that finds a negative relationship between ethnic diversity and social trust intuitively explains this with the idea that living amongst “ethnic others” creates anomie or social isolation, which erodes interpersonal trust both within and across ethnic groups [2, 6, 13–14]. In Putnam’s own words, Americans living in heterogeneous communities tend to “hunker down”, withdrawing from public and social life [2].

More recent work has proceeded to qualify the negative relationship between ethnic diversity and trust, often challenging the argument that ethnic diversity *per se* has detrimental effects on social cohesion. In particular, recent analyses have highlighted the importance of two *compositional* effects. First, ethnically diverse communities are not simply characterized by different patterns of intra and inter-group interactions, but are also composed of different ethnic groups with potentially different baseline levels of trust, which may in turn lead to lower *average* trust in a neighborhood. An example of how ethnoracial composition may influence the relationship between diversity and trust comes from a reanalysis of Putnam’s [2] data, in which Abascal and Baldassarri [13] show that the negative association between ethnic diversity and self-reported trust is an artifact of non-whites’ lower baseline levels of trust, coupled with their overrepresentation in heterogeneous neighborhoods. Once one takes into account that whites have higher levels of trust than Latinos and blacks, and that diverse neighborhoods have fewer whites, the supposedly negative effect of ethnic diversity disappears. Similar ethnoracial compositional effects have also been found in Europe [15–17].

Moreover, ethnically heterogeneous areas also tend to be poorer, and thus concentrated economic disadvantage, rather than ethnoracial diversity, might be at the basis of their lower cooperative capacity [13, 18–20]. The concentration of poverty in highly diverse areas is not just a common phenomenon in the United States but also in Europe [11, 21–26]. In the German context, too, individuals of “migration background” tend to live in neighborhoods that are both ethnically more diverse and poorer than comparable adjacent neighborhoods [22]. In fact, most studies on ethnic diversity and social capital control for income or unemployment status in their analyses at the individual and/or neighborhood level, albeit typically in order to avoid reporting spurious effects ([12], p. 467). Studies that explicitly compare the effects of ethnic heterogeneity and socioeconomic deprivation are rare but have shown that the negative effects of economic deprivation outweigh those of ethnic diversity in the UK [24, 27].

In this paper, we address the effects of two types of poverty–community level and individual–which we link to new empirical evidence from the experimental social sciences that suggests that poor people tend to have lower levels of trust [28–29] or are less trustworthy [30]. Importantly, if poor people tend to live in heterogeneous neighborhoods, it becomes critical to decouple the alleged “effect” of ethnic diversity from that of poverty in undermining trust and public goods provision. New behavioral evidence from trust experiments have shown that higher-class individuals in Germany are more trusting and trustworthy in an economic game when interacting with a stranger than lower social class individuals [28] Similarly, in a city-wide experiment on trust discrimination among inhabitants of Zurich, Falk and Zehnder [29] found that economic status was key for a district’s reputation and that people differentiate their investments in the trust experiment systematically depending on where the recipient lives by sending higher amounts if the receiver lives in a high-income district. However, Ermisch and Gambetta ([31], p.373) who examine the link between people’s self-reported financial situation and trust as measured in a trust game in the UK caution that the relationship may not always be linear as they find a U-shape relationship: people trust more when either they feel they would not lose much or when they have nothing to lose.

Part of the reason explaining the emerging experimental evidence suggesting that poor people display lower levels of altruism and trust could be that such prosocial behavior is crowded out by financial pressures, which are more pressing upon the poor, and which lead poor people to be more opportunistic [32–33]. Recent behavioral research also advances the idea that poverty produces a specific mind-set [34–36]. In particular, this scholarship suggests that people who are subjected to the stress of poverty suffer from higher cognitive load and tend to discount the future more than people who do not live in conditions of chronic disadvantage. These effects may lead poor individuals and communities to display lower levels of trust and cooperation. Since poor people tend to interact with other poor people, any effect linking poverty to lower rates of cooperation would be multiplied in poor communities. In sum, poverty, as well as contextual diversity, may be related to lower levels of cooperation.

### Measurement improvements

In this paper, we also improve on previous scholarship on the ethnic diversity debate by examining evidence from a reliable behavioral measure of trust–an incentivized trust game–embedded in the German Socio-Economic Panel Survey (GSOEP) in 2003–2005.

The vast majority of research on ethnic diversity and social trust is based on survey measures of generalized trust and in some cases particularized trust, such as trust in neighbors (e.g. [37]). However, scholars have raised concerns about attitudinal measures of trust, which often suffer from a range of shortcomings, including that it is unclear whom respondents imagine when asked about their general level of trust [38–41]. As a consequence, the interpretation of the question widely differs across individuals and societies (see also [38]). This makes the general trust question especially problematic as a reliable measure of social trust in studies interested in ethnic diversity and outgroup trust [13, 42]. Secondly, the most common formulation of the self-reported attitudinal measure of trust (“Generally speaking, would you say that most people can be trusted or that you can’t be too careful in dealing with people?” Response options are usually: (1) most people can be trusted / (2) one can’t be too careful) does not define the matter over which trust is to be exchanged: for instance, trusting someone to look after one’s child is different from trusting someone to return a lost wallet. The first reflects a judgment about another person’s competence and reliability, while the second concerns a belief about honesty. Thirdly, the self-reported measure of trust is subject to “social desirability bias”: since trust is commonly perceived to be a desirable characteristic [43], respondents may report themselves to be more trusting than they actually are. To address these issues, our analysis uses an anonymous trust game as a behavioral measure of trust. In this way, the social desirability bias is reduced through the use of monetary incentives. More importantly, both the object of trust (i.e. money) as well as the trust radius (i.e. other GSOEP respondents) are clearly defined, thus producing a “less-noisy” measure of trust. We elaborate more on our data and methods below.

Behavioral measures of trust have only been used in few studies on the effects of ethnic diversity. Exceptions are field experiments, such as Koopmans and Veit’s [22] “lost letter” experiment in Berlin and a replication study in the Netherlands [44], inspired by Sampson’s research in Chicago [20]. Other evidence using behavioral games stems from research on ethnic diversity in the African context [45], on intergroup discrimination after ethnic violence in postconflict societies [46] and from research on the intra-Jewish cleavages in Israel [47], although this work is based on different behavioral measures, such as the dictator game and public goods games. One study on the effects of immigrant-related ethnic diversity in Europe that also uses the results of a trust game is the recent work of Cettolin and Suetens [48]. However, differently from our study, which focuses on trusting behaviour, their research design focuses on trustworthiness rather than trust, finding that Dutch respondents reciprocate less if the trustor (Player 1) in a trust game has an immigrant name rather than a typical Dutch name.

In addition to using a behavioral measure from a representative sample of the entire residential population in Western Germany and comparing it to a standard attitudinal trust question, we complement the GSOEP data with new fine-grained information on the ethnic and economic composition of local areas (1x1km raster) contained in the Microm-RWI dataset [49]. This allows us to calculate indicators of local ethnic diversity and neighborhood socio-economic status at the level of the local postal code. Our data is therefore rich in terms of individual-level as well as community-level indicators of ethnic diversity and poverty. The advantage of this granularity is that, conceptually, many of the determinants of social trust are likely to arise at a neighborhood level. As Schaeffer ([11], Ch. 2) argues in his meta-analysis, the accuracy of heterogeneity measures and the size of the context are vital to detecting a relationship between ethnic diversity and trust: assuming that neighborhood composition is a good proxy of people’s overall exposure to interethnic others, the more local the measurement is, the better it would be at capturing underlying social dynamics. Dinesen and Sønderskov [50] provide empirical support for this argument by demonstrating in their study on ethnic diversity in Denmark that a negative relationship between ethnic diversity and trust only exists in small contexts (with a radius up to 80 meters) but not in aggregate contexts. Accordingly, we get as local as we can in measuring ethnic diversity by computing it at the zip code level, thus improving over previous analysis of the German SOEP which measured ethnic diversity at the administrative districts (*Kreis*) level [6].

While the use of such fine-grained data is common in other countries [8, 23, 44], studies on ethnic diversity in Germany usually had to resort to more aggregate measures (e.g. [51]). Furthermore, we believe that by combining a behavioral measure of trust with fine-grained neighborhood data on ethnic diversity and the rich information about individual-level and context-level characteristics of participants who stem from a representative section of the German population, we are able to contribute meaningfully to previous empirical work on neighborhood ethnic diversity and social trust. To the best of our knowledge, a similar design using a representative sample and a behavioral trust game has only been implemented at a much smaller scale in the UK [31] and the Netherlands [48, 52].

### The effects of diversity in comparative perspective

Inspired by earlier, U.S.-based studies [1–2], European scholars have investigated the relationship between ethnic diversity and trust in a variety of countries, often reaching less dramatic conclusions than their American counterpart (for a review, see [9]). In general, one might expect to find differences across continents, due to the substantial differences between the ethnic diversity that characterizes several European countries and the ‘entrenched’ discrimination that has characterized black-white relationships in the U.S., as well as institutional differences in terms of integration policies. Replication is therefore essential to test the generalizability of certain findings and determine the scope conditions of the underlying theories.

In this respect, Germany is a very interesting case study. In Germany immigration is a rather new phenomenon, which mostly occurred in the last 50 years. Moreover, in contrast to the idea of a multicultural “melting pot” where cultural minorities are recognized and accommodated, in Germany integration has much more heavily relied on assimilationist policies [51, 53]. Finally, in Germany, as in many other European countries, the ethnic divide partially overlaps with a Muslim-Christian divide, increasing the cultural distance between some ethnic groups and the mainstream population (e.g. on France see [54]).

Extant scholarship on the German case has so far produced mixed results. For example, while some scholars find a significant negative correlation between ethnic diversity and self-reported trust attitudes [55–56], others report null results [6] or find that ethno-cultural diversity in German cities does not exert the same negative effects on generalized and outgroup trust as documented in North America but that intergroup contact importantly moderates the effects of neighborhood diversity [57]. Using a “lost letter” experiment, Koopmans and Veit [22] find that fewer letters were returned from ethnically diverse neighborhoods in Berlin (suggesting a negative diversity effect), but that participants did not discriminate between letters addressed to German vs. Turkish cultural organizations (suggesting the absence of negative attitudes towards the out-group).

## Materials and methods

The GSOEP is a large annual household panel comprising a representative sample of members of German households 16 years or older [58]. In 2003, it contained 22,611 individuals in 12,061 households. Over a three-year period from 2003 to 2005, the GSOEP included a trust game based on Berg et al.’s design [59]. All experimental participants had been interviewed in the previous three waves of the GSOEP (since 2000) and were thus familiar with the survey organization and the particular interviewer who came to their home to conduct the experiment. This prior contact between the GSOEP enumerators and the participants of the behavioral game likely increased the credibility of the trust game scenario.

### The GSOEP trust game

#### Participants

This trust game was administered to a randomly selected subgroup of 1,315 people of the existing GSOEP panel, of which 658 played in the role of the trustor (Player 1). The overall participation rate was 95,2%. There was also a second smaller sample of 117 participants who played a trust game with 100 EUR instead of 10 EUR. To ensure comparability, we only look at the sample that received 10 EUR. We focus on the subsample of Western German participants, as the simultaneous analysis of individual- and context-level socio-economic status and ethnic diversity is only fully plausible in this context. Due to its history as part of the communist bloc, Eastern Germany did not see the high levels of immigration that have shaped the demographic profile of Western Germany. As a result, not only is the structural relationship between diversity and trust likely to be different in the East, but there is also very little variation in terms of neighborhood diversity in the East German sample. Indeed, ethnic diversity in the Eastern part of Germany is much smaller than in the West (i.e. less than 1% of the GSOEP trust game participants in the East are foreign citizens versus 6.5% in the West), thus creating problems of convergence in the statistical analysis. Our final sample therefore consists of 551 Western German individuals who were Player 1 (i.e. the “truster”) in the trust game in the years 2003 to 2005, resulting in 1,483 observations in total.

#### Procedure

The GSOEP implemented a version Berg et al.’s trust game (also known as investment game) [59], which was developed by Fehr et al. [60]. The basic structure of the game is the following: respondents were either assigned to the role of Player 1 or Player 2 (also known as the ‘trustee’) for the entire three years of the experiment. Both players received 10 Euros as starting capital each year and must decide how much of this amount to send to an anonymous other participant of the GSOEP. The choice they faced was to either keep all the money to themselves or to allocate some (any amount from 0–10 Euros) to the other player. The researcher team would then double whatever amount Player 1 allocated to Player 2 before Player 2 made his/her decision. Player 2 was told how much money he / she had received from Player 1, and could then decide to return all, parts or none of the money received to Player 1. The fact that Player 2 would receive this information was common knowledge to both players. Therefore, the amount passed by Player 1 is said to capture trust, ‘‘a willingness to bet that another person will reciprocate a risky move (at a cost to themselves),” and the amount returned from Player 2 to Player 1 reflects trustworthiness ([61], p. 85). Since we are here interested in general trust, which is important for the development of social capital, we focus our analysis on the behavior of Player 1 only.

In our sample of Western Germans, Player 1 sent on average 5.44 EUR (SD = 2.58) while Player 2 returned 4.90 EUR (SD = 2.67). The distribution of amounts passed by Player 1 in the trust game for the three consecutive years (2003–2005) is shown in the Supporting Information (see S1 Fig). To determine the final payoffs of Player 1, she/he was alphabetically matched with one Player 2. Although participants were told that they were assigned to another anonymous participant, they actually played with a fictional partner. According to the authors who designed and implemented the game in the GSOEP, this procedure was necessary because of the requirements related to representative sampling in a large panel [58]. A pre-test was conducted in 2002 to determine the amount that Player’s 2 received in the first round of the trust game in 2003. To control for interviewer bias, the interviewers surveyed either only participants who were assigned to be Player 1 or Player 2 throughout the three-year period. All participants received their individual payoff by mail (mean = 18.72 EUR, SD: 24.20, Min: 0, Max: 30).

#### Individual-level variables

Of the various available indicators of individual economic status, we use respondents’ reported household income in Euros. This individual-level economic indicator is the easiest to interpret but alternative measures, such as a person’s ‘objective social class’ (an index based on education, occupational prestige, and income) lead to substantively equivalent results. In addition, we include in all our regression models demographic control variables that could affect levels of trust, namely gender, age, educational attainment and residential stability [62–64].

#### Context-level variables

In order to determine the ethnic diversity and socio-economic status of the local context in which GSOEP respondents live, we use data from the Microm-RWI raster-dataset [49]. The Microm-RWI data provides information on purchasing power and ethnic composition at the level of 1x1km grid raster cells for the whole of Germany. This exceptionally fine-grained data allows us to calculate indicators of the ethnic fractionalization and relative wealth for all zip code areas in Germany. The zip-code level is the most precise geo-information available through the GSOEP for locating participants’ place of residence. The RWI-Microm data is available from 2005 onwards only, i.e. the last year during which the trust game was played. We therefore use the 2005 values as contextual measures for observations from all three years, under the assumption that neither the ethnic composition, nor the socio-economic status of a local area have changed dramatically in the course of these three years. During this three–year period, 94% of the sample remained living in the same apartment and 78% even reported to have lived in that same residence for 10 years or longer.

RWI-Microm provides data on the ethnic composition of households, divided into 12 different categories of ethno-linguistic origin. We use the share of individuals with non-German names in a given zip-code area as our indicator of *neighborhood ethnic diversity* and consider it a proxy of out-group exposure since the large majority of our respondents are German. Moreover, in the case of Germany, this measure is highly correlated with the Herfindahl index of ethnic fractionalization (see S3, S4 and S5 Tables in the Supporting Information) commonly used in similar studies on neighborhood ethnic diversity effects [10], and we therefore opted for the share of non-German named households for ease of interpretability. The ethnicity information is based on the analysis of the last name of the head of the household. It is thus a measure of ethnic *origin* of a family that does not take into account whether individuals are German citizens or not, or how long a person has lived in Germany. Since most migrants to Germany arrived in the last 50 years and differences between the native population and migrants are still perceived as significant in most cases, we believe that this measure is able to capture the multicultural character of a neighborhood.

Information on purchasing power is based on a combination of a large number of variables such as households’ employment status, age structure, car ownership and online shopping behavior. We use the average purchasing power per person in a zip-code area in Euros as our indicator for the socio-economic status of a participant’s local context. Note that the individual sources of information and the formula used to calculate the average purchasing power per person are proprietary. According to Microm, around 1 billion data points are used to calculate the purchasing power of the about 40.7 million households in Germany, i.e. an average of about 25 data points per household. Finally, all models also include the number of inhabitants in a zip-code area. To facilitate replication, we provide the code we used for our statistical analyses in the Supporting Information (see S2 Fig).

We would like to briefly also discuss some data limitations. First, some scholars have shown that the effect of diversity is moderated by the frequency and quality of interethnic contact [57, 65]. We unfortunately lack data on interethnic friendships and acquaintances for the participants in the trust game to take into account such variation in intergroup contact. Second, some scholars have pointed out that the effect of ethnic diversity on intergroup relations may not be linear but instead depend on a number of factors, such as the perceived diversity level [66]. For example, in Finland, research has found that majority group members in contexts with moderate levels of objective diversity but subjective perceptions of high levels of diversity will report greater negative outgroup trust than Finnish majority members in low- or high-diversity context [66]. We acknowledge that subjective perceptions are important but, unfortunately, such measures are also unavailable in the GSOEP data and we therefore rely on a measure of objective ethnic diversity as our contextual measure.

## Results

Table 1 provides an overview of the main variables of interest. As for the diversity measures, 6.5% of respondent in our sample are non-German citizens, and the share of individuals with non-German names in the zip-code where trust game participants live ranges between 1.4% and 32.4%, with a mean of 7.5%. As for our measures of socio-economic status, annual income ranges between 2,501€ for the poorest individual in the sample, and 67,866€ for the richest, with a mean of 17,859€. Average purchasing power at the zip-code level is 19,627€, with a minimum of 11,079€ and a maximum of 36,350€.

**Table 1:** Descriptive statistics for Player 1’s in trust game in GSOEP 2003–2005.

	Mean	SD	Min	Max	Obs
Amount sent by P1 in the trust game [GSOEP, 2003–2005]	5.44	2.58	0	10	1,483
Foreign citizen [GSOEP, 2003–2005]	0.065	0.25	0	1	1,483
Percent households with non-German names [Microm-RWI, 2005]	7.54	4.47	1.44	32.34	551
Individual income in € for German citizens [GSOEP, 2003–2005]	18,792	8,631	2,501	67,866	1,387
Individual income in € for foreign citizens [GSOEP, 2003–2005]	12,246	6,326	2,700	30,132	96
Purchasing power in zip code in € [Microm-RWI, 2005]	19,627	3,571	11,079	36,350	551

In order to estimate the simultaneous impact of the variables and to properly accommodate the data structure, we use multilevel modeling. Since in our data, observations are nested within individuals, we allow intercepts to vary by individuals. The model can be written as

$$y_{ji}=\alpha +X_{ji}\beta +\mu_{j}+\varepsilon_{ji}$$

where y is the amount sent in the trust game, j stands for individuals, i for individual observations and the vector **X** contains the independent and control variables. The additional error term μ indicates that the intercept is estimated separately for each individual, and ε is the error term associated with individual observations. μ and ε are assumed to be uncorrelated. α is the overall intercept. All models include a dummy indicating the survey year. Maximum likelihood estimation is used to fit the models.

Note that we also run our model with an additional zipcode level random effect. Results remain substantively unchanged. In addition, we also run a model with individual-level fixed effects. However, we note that the trust game was only implemented in three consecutive years (2003–2005), and therefore the vast majority of respondents experienced only very small changes with respect to income, diversity exposure, etc. over this short period. Consequently, our fixed effects estimates lose a substantial amount of precision. For this reason, we stick with the random effects model.

Regression results are presented in Table 2. We do not display the results for the independent and zip-code level controls in the main manuscript, but full regression tables can be found in the Supporting Information (S1 Table). Furthermore, in order to exclude the possibility that the results are driven by the small number of non-Germans in the sample–only 6.5% of respondents are foreign citizens—in S2 Table in the Supporting Information, we present results for models (2–6) for the German citizen sample only. We find that our results remain substantively unchanged when excluding foreign citizens from the sample. Unfortunately, a lack of data on the ethnic composition of neighborhood diversity and the small number of foreign citizens in the sample, for whom higher levels of ethnic diversity in the neighborhood not necessarily mean a greater share of ethnic ingroup-members, do not allow us to meaningfully examine the effects of neighborhood diversity for immigrants’ social trust.

**Table 2:** Behavioral trust conditional on individual- and zip-code-level indicators of socio-economic status and ethnic diversity.

	(1)	(2)	(3)	(4)	(5)	(6)
	Behavioral trust	Behavioral trust	Behavioral trust	Behavioral trust	Behavioral trust	Behavioral trust
Foreign citizen	-0.64* (0.35)	—	—	—	-0.42 (0.35)	—
% households with non-German names	—	-0.05** (0.02)	—	—	-0.05** (0.02)	-0.05** (0.02)
Income in 10,000€	—	—	0.34*** (0.09)	—	0.32*** (0.09)	0.34*** (0.09)
Purchasing power in zip code in 10,000€	—	—	—	0.22 (0.25)	0.18 (0.25)	—
% hh with non-German names BY Income in 10,000€	—	—	—	—	—	-0.02 (0.02)
Constant	5.05*** (0.44)	5.27*** (0.46)	4.64*** (0.44)	4.60*** (0.59)	4.75*** (0.59)	5.19*** (0.44)
Individual and zip-level controls	Yes	Yes	Yes	Yes	Yes	Yes
Survey year indicators	Yes	Yes	Yes	Yes	Yes	Yes
Observations	1,483	1,483	1,483	1,483	1,483	1,483
Individuals	551	551	551	551	551	551
Rho/ICC	.45	.45	.44	.45	.43	.43

Standard errors in parentheses

* p<0.1

** p<0.05

*** p<0.01

Models 1–4 of our main results in Table 2 estimate the bivariate relationships between behavioral trust and each single predictor of interest using the fully specified model. Foreign nationals are less trusting than German nationals, giving on average 0.64€ (12%) less to an anonymous other in the trust game (column 1). The percentage of households headed by individuals with non-German names, our measure of neighborhood diversity, is negatively associated with behavioral trust. Moving from a relatively homogenous neighborhood with 5% foreign names to one with 15% foreign names is associated with a 0.46 € drop in contributions (column 2).

**Fig 1:**
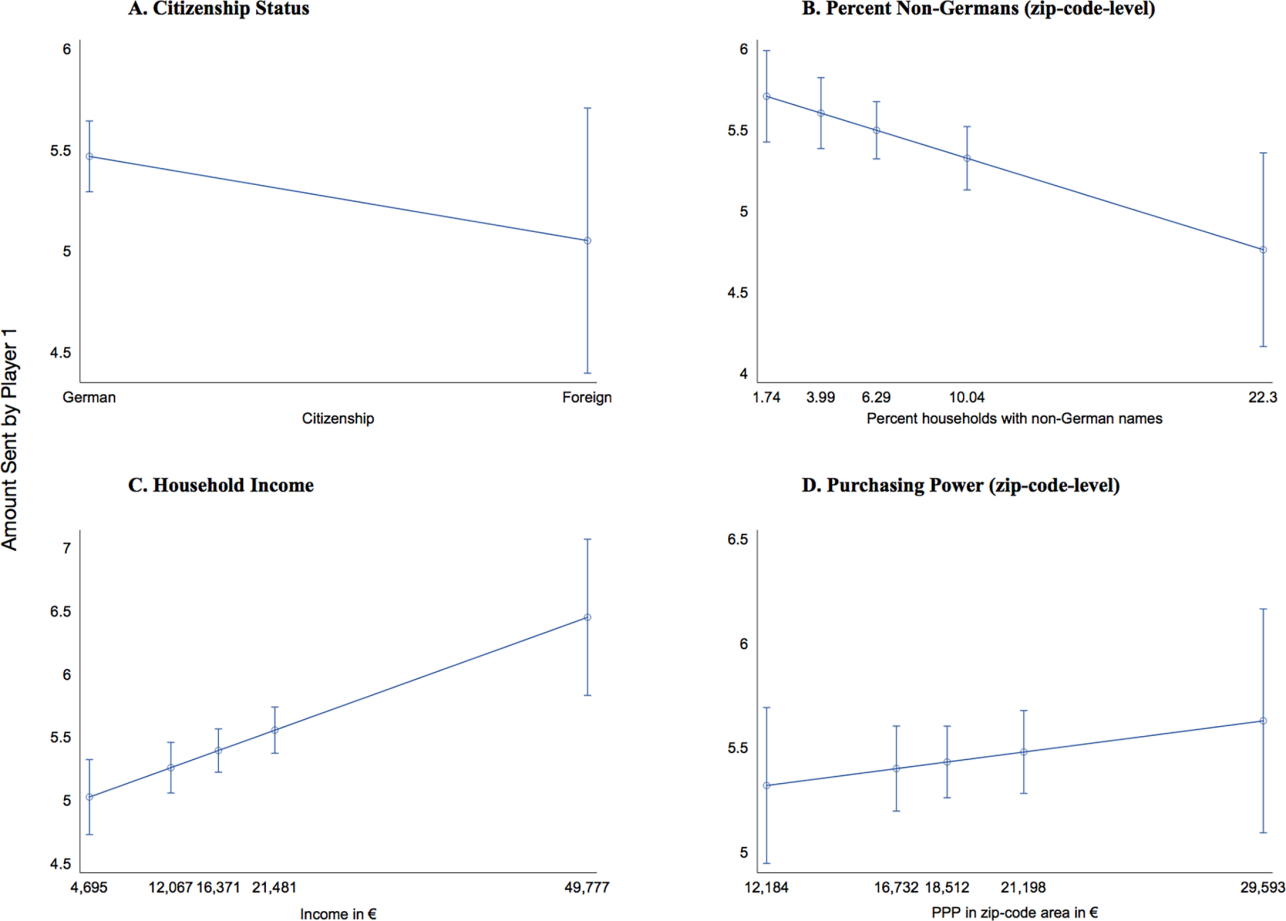
Predicted values for behavioral trust for individual- and zip-code-level indicators of socio-economic status and ethnic diversity. (A) Citizenship Status, (B) % non-Germans (zip-code level), (C) Household Income, (D) Purchasing Power (zip-code level) for the 1%, 25%, 50%, 75% and 99% percentile (full models with controls).

Individual-level income strongly and positively predicts trust. A 15,000 € difference in income is associated with a 0.52 € increase in behavioral trust (column 3). Purchasing power in the participants’ zip-code area of residence is also positively related to trust, but this estimate is not statistically significant (column 4). Fig 1A to Fig 1D represent all effects graphically, plotting predicted values for behavioral trust against the 1%, 25%, 50%, 75% and 99% percentile of the variables of interest.

Next, given our focus on compositional effects, we report results from a model in which all four variables of interest are introduced in the model simultaneously (columns 5). At the individual level, the predictive capacity of income remains stable, while immigrant status becomes non-significant. This is easily explained: foreign citizens tend to be poorer, and, once their economic conditions are factored in, being an immigrant does not make them less likely to trust others. Moving to consider contextual variables, the coefficient for neighborhood-level diversity remains significant at the p < .05 level, while neighborhood socio-economic condition remains non significant.

We also consider a model including the interaction between individual income and neighborhood diversity (column 6). According to ethnic competition theories, low-income individuals may be more threatened by immigration, either due to fears of economic competition and / or beliefs about immigrants’ burden on the welfare system. We would therefore expect that the effect of neighborhood diversity would be largest amongst low-income individuals. Interestingly, the interaction is non-significant, leading us to conclude that the paths through which individual economic conditions and neighborhood diversity are related to social trust in Western Germany are likely to be independent (in statistical parlance, their ‘effect’ is additive).

In Table 3, we show how the behavioral measure of trust performs in our analysis compared to a standard attitudinal measure of trust, in which participants were asked whether they agreed that ‘on the whole, most people can be trusted’. Column 1 reports standardized coefficients for the behavioral measure of trust using all three years of data available. As already reported in column 6 of Table 2, individual income is the strongest predictor of trusting behavior, followed by neighborhood diversity.

**Table 3:** Comparing behavioral and attitudinal trust conditional on individual- and zip-code-level indicators of socio-economic status and ethnic diversity.

	(1)	(2)	(3)
	Behavioral trust 3 years (Std)	Behavioral trust 1 year (Std)	Attitudinal trust 1 year (Std)
Foreign citizen	-0.10 (0.08)	-0.16 (0.11)	0.00 (0.03)
% households with non-German names	-0.21** (0.10)	-0.10 (0.11)	0.01 (0.03)
Income in 10,000€	0.31*** (0.09)	0.21 (0.13)	0.06* (0.03)
Purchasing power in zip code in 10,000€	0.02 (0.10)	0.06 (0.11)	-0.02 (0.03)
Constant	4.66*** (0.44)	5.09 *** (0.58)	2.41*** (0.15)
Individual and zip-level controls	Yes	Yes	Yes
Survey year indicators	Yes	Yes	Yes
Observations	1,483	538	538
Individuals	551	538	538
Rho/ICC	.43		

Standard errors in parentheses

* p<0.1

** p<0.05

*** p<0.01

Since the attitudinal trust measure is only available for one year, we repeat the analysis of the standardized behavioral measure with a sub-sample for which we also have the attitudinal measure. Results in column 2 of Table 3 show that while the coefficients lose statistical significance at conventional levels, the magnitude of the income coefficient changes little. Finally in column 3 of Table 3, we report estimates for self-reported attitudinal trust. We find that while the model more precisely estimates the effect of income, the predictive capacity of our four variables of interest, as well as of the model in its entirety, appears to be lower compared to the one-year model predicting behavioral trust (column 2), however, differently from model 2, we find that the income estimate is marginally significant. This result is also consistent with previous research using the GSOEP attitudinal trust questions which found no significant negative effect of ethnic diversity at the district (Kreis) level on self-reported levels of trust [6]

## Conclusion

This article investigates the relationship between ethnic diversity, poverty and social trust in Germany. We expected to find a negative relationship between diversity and trust, and hypothesized that this relationship could be explained, at least in part, by two compositional effects related to the ethnicity and poverty of the people who live in diverse neighborhoods. Our first major result is that poverty is indeed significantly and negatively related to social trust. While many previous studies have controlled for economic conditions at the individual and neighborhood level (e.g. [2, 27, 50), relatively little emphasis has been given to the potential compositional effect of socio-economic disadvantage in impeding social trust in ethnically diverse communities (but see e.g. [24, 27, 67]). In adding to this literature, we also add to the growing scholarship on the detrimental effects of poverty on individual behavior [32, 34–36].

Second, we find that after controlling for income, foreign citizens and Germans are equally trusting. This is in contrast with findings from Abascal and Baldassarri [13], who showed that, in the United States, the alleged negative effect of diversity can be simply explained as an artifact of the ethnic composition of diverse neighborhoods. Fundamental differences between the minority groups and the nature of majority-minority relations may be at the basis of these country-level differences. Alternatively, the difference could also be due to a small and heterogeneous sample of foreign citizens, which does not allow us to examine in depth the relationship between neighborhood diversity and social trust for different ethnic groups or for foreign citizens across a spectrum of socio-economic status.

Finally, while the proportion of neighbors with immigrant background, is neither the only nor the most powerful predictor of social trust, this indicator remains significant even after controlling for respondents’ immigration status and household income. In fact, the coefficient of neighborhood ethnic diversity remains substantively unchanged when controlling for individual level confounders. Our results thus suggest that social cohesion in Germany, is related not only to individual and household characteristics, but also to characteristics of the immediate neighborhood (cp. [17]).

This study has a number of limitations that can be addressed in future work. First, the data used have been collected 15 years ago, and we therefore do not know whether current patterns are different given the recent increase immigrant-related ethnic diversity in Germany. We could benefit from more recent data, especially if the same behavioral measure was used again and the study would include many of the same respondents. Furthermore, more recent data would also be good, in that it would allow us to test for changes over time. In our short time span (2003–2005), the explanatory variables did not change much. However, with more recent data we could conduct a longitudinal test of the impact of neighborhood and individual level variables applying fixed effects models, similar to recent work in the UK using the British Household Panel Survey [3].

These limitations aside, our results indicate that there is a significant correlation between a certain type of contextual diversity, the proportion of immigrants in the neighborhood, and a behavioral measure of social trust (but not a survey measure of self-reported trust). Thus, our results echo previous studies using different outcome measures in the German context [22, 55–56] that also find a negative relationship between outgroup exposure and social trust. Moreover, we find that the ethnic composition of a community is more tenuously related to social trust than the economic disadvantage of its members. This is especially important given what we know about the concentration of poverty in highly diverse areas in Germany and elsewhere in Europe [11, 22, 24, 67]. This result calls for more careful theorizing about precisely why living amongst poor(er) ethnic minorities should erode trust: are individuals reacting to the economic circumstances of their neighbors, their immigration background, or a combination of both factors?

This scholarly debate also has important policy implications, especially in the light of the recent waves of immigration to European countries, and the rise in support for populist parties and anti-immigration policies in Europe and the United States. For policymakers, our results imply that it is important to have a good understanding of the different compositional as well as contextual channels that underlie lower levels of trust. Furthermore, we echo previous scholars in their recommendations that policymakers aiming at improving social cohesion in multi-ethnic societies should pay more attention to improving the lives of those who are economically disadvantaged [24, 67]. To this end, future research can inform better policies by investigating the causal mechanisms linking poverty to lower levels of social trust.

## Supporting information

S1 Fig Distribution of amounts sent by Player 1 in the GSOEP trust game. (TIFF)

S2 Fig Replication file for analyses and graphs. (PDF)

S1 Table Behavioral trust conditional on socio-economic status and ethnic diversity with individual and zip-code level controls, full models (5–6 only). (DOCX)

S2 Table Behavioral trust conditional on individual- and zip-code-level indicators of socio-economic status and ethnic diversity (for GERMAN citizens only). (DOCX)

S3 Table Ethnic diversity indicators at the Kreis-level for all years (2003–2005). (DOCX)

S4 Table Ethnic diversity indicators by survey year. (DOCX)

S5 Table Correlations between ethnic diversity indicators at district-level. (DOCX)

## References

[1] Alesina A, La Ferrara E. Participation in heterogeneous communities. The quarterly journal of economics. 2000 8 1;115(3):847–904.

[2] Putnam RD. E pluribus unum: Diversity and community in the twenty‐first century the 2006 Johan Skytte Prize Lecture. Scandinavian political studies. 2007 6 1;30(2):137–74.

[3] Laurence J, Bentley L. Does ethnic diversity have a negative effect on attitudes towards the community? A longitudinal analysis of the causal claims within the ethnic diversity and social cohesion debate. European Sociological Review. 2015 8 24;32(1):54–67.

[4] Demireva N, Heath A. Diversity and the civic spirit in British neighbourhoods: an investigation with MCDS and EMBES 2010 Data. Sociology. 2014 8;48(4):643–62. 10.1177/0038038513516695 25544783 PMC4275549

[5] Koopmans R, Veit S. Ethnic diversity, trust, and the mediating role of positive and negative interethnic contact: A priming experiment. Social science research. 2014 9 1;47:91–107. 10.1016/j.ssresearch.2014.03.014 24913947

[6] Levels M, Scheepers P, Huijts T, Kraaykamp G. Formal and informal social capital in Germany: The role of institutions and ethnic diversity. European Sociological Review. 2015 9 2;31(6):766–79.

[7] Uslaner EM. Segregation and mistrust: Diversity, isolation, and social cohesion. Cambridge University Press; 2012 9 17.

[8] Tolsma J, van der Meer TW. Losing wallets, retaining trust? The relationship between ethnic heterogeneity and trusting coethnic and non-coethnic neighbours and non-neighbours to return a lost wallet. Social indicators research. 2017 3 1;131(2):631–58. 10.1007/s11205-016-1264-y 28366976 PMC5357475

[9] Dinesen PT, Sønderskov KM. Ethnic Diversity and Social Trust: A Critical Review of the Literature and Suggestions for a Research Agenda InOxford Handbook on Social and Political Trust 2017 (pp. 175–204). Oxford University Press.

[10] Schaeffer M. Can competing diversity indices inform us about why ethnic diversity erodes social cohesion? A test of five diversity indices in Germany. Social Science Research. 2013 5 1;42(3):755–74. 10.1016/j.ssresearch.2012.12.018 23521993

[11] Schaeffer M. Ethnic diversity and social cohesion: Immigration, ethnic fractionalization and potentials for civic action Ashgate Publishing, Ltd.; 2014 2 28.

[12] Meer TV, Tolsma J. Ethnic diversity and its effects on social cohesion. Annual Review of Sociology. 2014 7 30;40:459–78.

[13] Abascal M, Baldassarri D. Love thy neighbor? Ethnoracial diversity and trust reexamined. American Journal of Sociology. 2015 11 1;121(3):722–82. 10.1086/683144 26900618

[14] Gesthuizen M, Van der Meer T, Scheepers P. Ethnic diversity and social capital in Europe: tests of Putnam's thesis in European countries. Scandinavian Political Studies. 2009 6 1;32(2):121–42.

[15] De Vroome T, Hooghe M, Marien S. The origins of generalized and political trust among immigrant minorities and the majority population in the Netherlands. European Sociological Review. 2013 6 18;29(6):1336–50.

[16] Gijsberts M, Van Der Meer T, Dagevos J. ‘Hunkering down’ in multi-ethnic neighbourhoods? The effects of ethnic diversity on dimensions of social cohesion. European Sociological Review. 2011 3 26;28(4):527–37.

[17] Koopmans R, Schaeffer M. Relational diversity and neighbourhood cohesion. Unpacking variety, balance and in-group size. Social science research. 2015 9 1;53:162–76. 10.1016/j.ssresearch.2015.05.010 26188445

[18] Sampson RJ, Raudenbush SW, Earls F. Neighborhoods and violent crime: A multilevel study of collective efficacy. Science. 1997 8 15;277(5328):918–24. 9252316 10.1126/science.277.5328.918

[19] Sampson RJ, Groves WB. Community structure and crime: Testing social-disorganization theory. American journal of sociology. 1989 1 1;94(4):774–802.

[20] Sampson RJ. Great American city: Chicago and the enduring neighborhood effect. University of Chicago Press; 2012 2 15.

[21] Delhey J, Newton K, Welzel C. How general is trust in “most people”? Solving the radius of trust problem. American Sociological Review. 2011 10;76(5):786–807.

[22] Koopmans R, Veit S. Cooperation in Ethnically Diverse Neighborhoods: A Lost‐Letter Experiment. Political Psychology. 2014b 6 1;35(3):379–400.

[23] Lancee B, Dronkers J. Ethnic, religious and economic diversity in Dutch neighbourhoods: Explaining quality of contact with neighbours, trust in the neighbourhood and inter-ethnic trust. Journal of Ethnic and Migration Studies. 2011 4 1;37(4):597–618.

[24] Letki N. Does diversity erode social cohesion? Social capital and race in British neighbourhoods. Political Studies. 2008 3 1;56(1):99–126.

[25] Musterd S. Social and ethnic segregation in Europe: levels, causes, and effects. Journal of urban affairs. 2005 8 1;27(3):331–48.

[26] Musterd S, Marcińczak S, Van Ham M, Tammaru T. Socioeconomic segregation in European capital cities. Increasing separation between poor and rich. Urban Geography. 2017 8 9;38(7):1062–83.

[27] Sturgis P, Brunton-Smith I, Read S, Allum N. Does ethnic diversity erode trust? Putnam’s ‘hunkering down’thesis reconsidered. British journal of political science. 2011 1;41(1):57–82.

[28] Korndörfer M, Egloff B, Schmukle SC. A large scale test of the effect of social class on prosocial behavior. PloS one. 2015 7 20;10(7):e0133193 10.1371/journal.pone.0133193 26193099 PMC4507988

[29] Falk A, Zehnder C. A city-wide experiment on trust discrimination. Journal of Public Economics. 2013 4 1;100:15–27.

[30] Ermisch J, Gambetta D. Income and trustworthiness. Sociological Science. 2016 8 17;3:710–29.

[31] Ermisch J, Gambetta D. Do strong family ties inhibit trust?. Journal of Economic Behavior & Organization. 2010 9 1;75(3):365–76.

[32] Andreoni J, Nikiforakis N, Stoop J. Are the rich more selfish than the poor, or do they just have more money? A natural field experiment. National Bureau of Economic Research; 2017 3 9.

[33] Gambetta D, Hamill H. Streetwise: How taxi drivers establish customer's trustworthiness. Russell Sage Foundation; 2005 6 30.

[34] Banerjee AV, Duflo E. Poor Economics: Barefoot Hedge-fund Managers, DIY Doctors and the Surprising Truth about Life on Less Than 1 [dollar] a Day Penguin Books; 2012.

[35] Haushofer J, Fehr E. On the psychology of poverty. Science. 2014 5 23;344(6186):862–7. 10.1126/science.1232491 24855262

[36] Mani A, Mullainathan S, Shafir E, Zhao J. Poverty impedes cognitive function. science. 2013 8 30;341(6149):976–80. 10.1126/science.1238041 23990553

[37] Laurence J. Wider-community segregation and the effect of neighbourhood ethnic diversity on social capital: An investigation into intra-neighbourhood trust in Great Britain and London. Sociology. 2017 10;51(5):1011–33. 10.1177/0038038516641867 28989199 PMC5603975

[38] Freitag M, Bauer PC. Testing for measurement equivalence in surveys: Dimensions of social trust across cultural contexts. Public opinion quarterly. 2013 1 1;77(S1):24–44.

[39] Glaeser EL, Laibson DI, Scheinkman JA, Soutter CL. Measuring trust. The quarterly journal of economics. 2000 8 1;115(3):811–46.

[40] Ermisch J, Gambetta D, Laurie H, Siedler T, Noah Uhrig SC. Measuring people's trust. Journal of the Royal Statistical Society: Series A (Statistics in Society). 2009 10 1;172(4):749–69.

[41] Reeskens T. But who are those “most people” that can be trusted? Evaluating the radius of trust across 29 European societies. Social indicators research. 2013 11 1;114(2):703–22.

[42] Gundelach B. In diversity we trust: The positive effect of ethnic diversity on outgroup trust. Political Behavior. 2014 3 1;36(1):125–42.

[43] Rotter J. B. (1967). A new scale for the measurement of interpersonal trust. Journal of personality, 35(4), 651–665. 4865583 10.1111/j.1467-6494.1967.tb01454.x

[44] Volker B, Mollenhorst G, Steenbeek W, Schutjens V, Flap H. Lost letters in Dutch neighborhoods: A field experiment on collective efficacy. Social Forces. 2015 10 20;94(3):953–74.

[45] Habyarimana J, Humphreys M, Posner DN, Weinstein JM. Coethnicity: diversity and the dilemmas of collective action. Russell Sage Foundation; 2009 7 30.

[46] Whitt S, Wilson RK. The dictator game, fairness and ethnicity in postwar Bosnia. American Journal of Political Science. 2007 7 1;51(3):655–68.

[47] Enos RD, Gidron N. Intergroup behavioral strategies as contextually determined: Experimental evidence from Israel. The Journal of Politics. 2016 7 1;78(3):851–67.

[48] Cettolin E, Suetens S. Return on trust is lower for immigrants. The Economic Journal. 2017 8.

[49] Budde R, Eilers L. Sozioökonomische Daten auf Rasterebene: Datenbeschreibung der microm-Rasterdaten. RWI Materialien; 2014.

[50] Dinesen PT, Sønderskov KM. Ethnic diversity and social trust: Evidence from the micro-context. American Sociological Review. 2015 6;80(3):550–73.

[51] Koopmans R, Schaeffer M. Statistical and perceived diversity and their impacts on neighborhood social cohesion in Germany, France and the Netherlands. Social Indicators Research. 2016 2 1;125(3):853–83.

[52] Bellemare C, Kröger S. On representative social capital. European Economic Review. 2007 1 1;51(1):183–202.

[53] Koopmans R. Trade-offs between equality and difference: Immigrant integration, multiculturalism and the welfare state in cross-national perspective. Journal of ethnic and migration studies. 2010 1 1;36(1):1–26.

[54] Adida CL, Laitin DD, Valfort MA. Why Muslim integration fails in Christian-heritage societies. Harvard University Press; 2016 1 4.

[55] Traunmüller R. Moral communities? Religion as a source of social trust in a multilevel analysis of 97 German regions. European Sociological Review. 2010 3 6;27(3):346–63.

[56] Gundelach B, Traunmüller R. Beyond generalised trust: Norms of reciprocity as an alternative form of social capital in an assimilationist integration regime. Political Studies. 2014 10 1;62(3):596–617.

[57] Stolle D, Petermann S, Schmid K, Schönwälder K, Hewstone M, Vertovec S, Schmitt T, Heywood J. Immigration-related diversity and trust in German cities: The role of intergroup contact. Journal of Elections, Public Opinion & Parties. 2013 8 1;23(3):279–98.

[58] Wagner G, Frick J, Schupp J (2007) The German Socio-Economic Panel study (SOEP): Scope, evolution and enhancements. J Appl Soc Sci Stud 127: 139–169.

[59] Berg J, Dickhaut J, McCabe K. Trust, reciprocity, and social history. Games and economic behavior. 1995 7 1;10(1):122–42.

[60] Fehr E, Fischbacher U, von Rosenbladt B, Schupp J, Wagner GG. A Nation-Wide Laboratory. Examining trust and trustworthiness by integrating behavioral experiments into representative survey. Schmollers Jahrbuch. 2002;122(519):542.

[61] Camerer CF. Behavioral game theory: Experiments in strategic interaction. Princeton University Press; 2011 9 5.

[62] Sutter M, Kocher MG. Trust and trustworthiness across different age groups. Games and Economic Behavior. 2007 5 1;59(2):364–82.

[63] Ben-Ner A, Halldorsson F. Trusting and trustworthiness: What are they, how to measure them, and what affects them. Journal of Economic Psychology. 2010 2 1;31(1):64–79.

[64] Trautmann ST, van de Kuilen G, Zeckhauser RJ. Social class and (un) ethical behavior: A framework, with evidence from a large population sample. Perspectives on Psychological Science. 2013 9;8(5):487–97. 10.1177/1745691613491272 26173207

[65] Schmid K, Ramiah AA, Hewstone M. Neighborhood ethnic diversity and trust: The role of intergroup contact and perceived threat. Psychological science. 2014 3;25(3):665–74. 10.1177/0956797613508956 24434239

[66] Celikkol G, Mähönen TA, Jasinskaja-Lahti I. The interplay between objective and subjective ethno-cultural diversity in predicting intergroup relations. Journal of Ethnic and Migration Studies. 2017 7 4;43(9):1399–416.

[67] Laurence J. The effect of ethnic diversity and community disadvantage on social cohesion: A multi-level analysis of social capital and interethnic relations in UK communities. European Sociological Review. 2009 12 9;27(1):70–89.

