# Supplementary figures and images for: Ethnic diversity, poverty and social trust in Germany: Evidence from a behavioral measure of trust
Source: PLoS One. 2018 Jul 18;13(7):e0199834. doi: 10.1371/journal.pone.0199834 (PMC6051567; doi:10.1371/journal.pone.0199834)

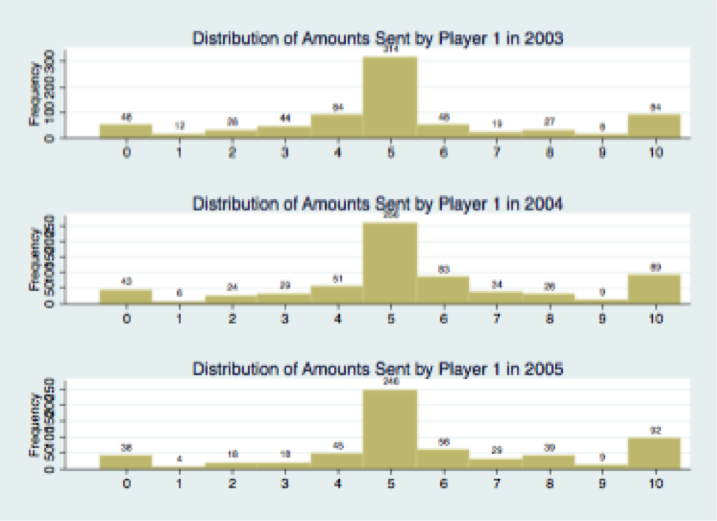

Supplement: S1 Fig — (TIFF) [file pone.0199834.s001.tiff]
