# Supplementary material for: Ethnic diversity, poverty and social trust in Germany: Evidence from a behavioral measure of trust
Source: PLoS One. 2018 Jul 18;13(7):e0199834. doi: 10.1371/journal.pone.0199834 (PMC6051567; doi:10.1371/journal.pone.0199834)
Supplement: S2 Fig — (PDF) [file pone.0199834.s002.pdf]

S2 Fig. Replication file for analyses and graphs

```
/* Information about two data sources:
access to the geo-referenced data of the German SOEP is only available in-house for visiting researchers
(requests should be send to soepmail@diw.de).
Postal-level measures of context-level ethnic diversity and purchasing power in Germany
are available for purchase from microm Consumer marketing(info@microm.de).  */

*----------*
* Prep
*----------*
cd "H:\"
log using soeplog270318, replace
set scheme burd7

* use "trust_march18.dta", clear
eststo clear
set more off
set matsize 5000
set linesize 255
xtset persnr
keep if player==1 & west==1 //Only look at player 1 behavior in Western Germany from GSOEP
* Sample variable: necessary because two zip-codes are not matched, and the model is otherwise estimated with varying numbers of observations

*---------------------------------------------------------------------------*
* Table 1 Descriptive statistics for Player 1's in trust game in GSOEP 2003-2005
*---------------------------------------------------------------------------*

sum pgive perc_nogermnames05 foreign_citizen plz_ppp logincome male age isced nevermoved inhabitants_zip svyyear
* Two obs lost because of unmatched zip-codes
* Full income data, but one dropped because here we use logincome, and one person reports an income of zero, which led to a missing value

```

```


* Multilevel model with random effects
*-----------------------------------
xtreg pgive foreign_citizen `controls', re mle nolog
eststo main1
xtreg pgive perc_nogermnames05 `controls', re mle nolog
eststo main2
xtreg pgive income `controls', re mle nolog
eststo main3
xtreg pgive plz_ppp `controls', re mle nolog
eststo main4
xtreg pgive foreign_citizen perc_nogermnames05 income  plz_ppp `controls', re mle nolog
eststo main5 // full model
xtreg pgive perc_nogermnames05 income c.cent_perc_nogermnames05##c.cent_income  `controls', re mle nolog
eststo main6 //interaction effect between HH income and % households with non-German names
esttab main1 main2 main3 main4 main5 main6 ,  nogaps wrap varwidth(30) compress se  b(%9.2f) width() scalars("N_g Individuals"  "rho Rho/ICC") sfmt(%9.2g) label star(* 0.1 ** 0.05 *** 0.01) indicate("Ind & zip-level controls = *male*" "Round indicators = *svyyear*") drop(age isced nevermoved inhabitants_zip) eqlabels(, none) replace nomtitle title(Behavioural trust conditional on individual and zip-code-level indicators of economic status and ethnic diversity, West Germany only)


*------------------------------------------------------------------------------*
* Table 3 Comparing behavioral and attitudinal trust
*------------------------------------------------------------------------------*

* Standardizing variables
egen std_perc_nogermnames05 = std(perc_nogermnames05)
egen std_foreign_citizen = std(foreign_citizen)
egen std_plz_ppp = std(plz_ppp)
egen std_income = std(income)

* Model 1: Behavioral trust 3 years (std.)
local controls i.male age isced nevermoved inhabitants_zip i.svyyear
xtreg pgive std_perc_nogermnames05 std_foreign_citizen std_plz_ppp std_income `controls', re mle nolog
eststo std1

* Model 2: Behavioral trust 1 year (std.)
```

```
margins, at(citizenship==(0 1)) atmeans
marginsplot, name(citizenship, replace) ytitle("") title("Citizenship Status")

* Figure 1b. % non-Germans (zip-code level)
margins, at(perc_nogermnames05==(1.74  3.99 6.29  10.04  22.30)) atmeans
marginsplot, name(perc_nogermnames05, replace) ytitle("") title("% non-Germans (zip-code level)")

* Figure 1c. Household Income
margins, at(income==(.4695 1.2067  1.6371 2.1481  4.9777)) atmeans
marginsplot, name(income, replace) ytitle("") title("Household income")

* Figure 1d. Purchasing power (zip-code level)
margins, at(plz_ppp==(1.2184 1.6732 1.8512  2.1198  2.9593)) atmeans
marginsplot, name(plz_ppp, replace) ytitle("") title("Purchasing power")



log close

translator set smcl2pdf pagesize custom
translator set smcl2pdf pagewidth 11.0
translator set smcl2pdf pageheight 8.5
translator set smcl2pdf logo off
translator set smcl2pdf fontsize 8
translator set smcl2pdf lmargin 0.4
translator set smcl2pdf rmargin 0.4
translator set smcl2pdf tmargin 0.4
translator set smcl2pdf bmargin 0.4
translate soeplog270318.smcl soeplog270318.pdf, replace
```
