# Supplementary material for: Ethnic diversity, poverty and social trust in Germany: Evidence from a behavioral measure of trust
Source: PLoS One. 2018 Jul 18;13(7):e0199834. doi: 10.1371/journal.pone.0199834 (PMC6051567; doi:10.1371/journal.pone.0199834)
Supplement: S1 Table — (DOCX) [file pone.0199834.s003.docx]

**S1 Table. Behavioral trust conditional on socio-economic status and ethnic diversity with individual and zip-code level controls, full models (5-6 only).**

|  | (5) | (6) |
| --- | --- | --- |
|  | Behavioral trust | Behavioral trust |
| Foreign citizen | -0.42  (0.35) | --- |
| % households with non-German names | -0.05**  (0.02) | -0.05**  (0.02) |
| Income in 10,000€ | 0.32***  (0.09) | 0.34***  (0.09) |
| Purchasing power in zip code in 10,000€ | 0.18  (0.25) | --- |
| % hh with non-German names BY Income in 10,000€ | --- | -0.02  (0.02) |
| Constant | 4.75***  (0.59) | 5.19***  (0.44) |
| Male | -0.23  (0.18) | -0.23  (0.18) |
| Age | -0.01**  (0.01) | -0.01**  (0.01) |
| Education | 0.16**  (0.07) | 16**  (0.07) |
| Residential Stability (not moved for the last 10 years) | 0.01  (0.20) | 0.01  (0.20) |
| Population density in zip code area | 0.08  (0.08) | 0.08  (0.08) |
| Survey year indicators | Yes | Yes |
| σ_ μ | 1.67***  (0.08) | 1.66***  (0.08) |
| σ_ ϵ | 1.89***  (0.04) | 1.89***  (0.04) |
| Observations | 1,483 | 1,483 |
| Individuals | 551 | 551 |
| Rho/ICC | .43 | .43 |

Standard errors in parentheses * p<0.1, ** p<0.05, *** p<0.01
