# Supplementary material for: Ethnic diversity, poverty and social trust in Germany: Evidence from a behavioral measure of trust
Source: PLoS One. 2018 Jul 18;13(7):e0199834. doi: 10.1371/journal.pone.0199834 (PMC6051567; doi:10.1371/journal.pone.0199834)
Supplement: S2 Table — (DOCX) [file pone.0199834.s004.docx]

**S2 Table. Behavioral trust conditional on individual- and zip-code-level indicators of socio-economic status and ethnic diversity (for GERMAN citizens only)**

|  | (2) | (3) | (4) | (5) | (6) |
| --- | --- | --- | --- | --- | --- |
|  | Behavioral trust | Behavioral trust | Behavioral trust | Behavioral trust | Behavioral trust |
| % households with non-German names | -0.04**  (0.02) | --- | --- | -0.04**  (0.02) | -0.05**  (0.02) |
| Income in 10,000€ | --- | 0.33***  (0.10) | --- | 0.31***  (0.10) | 0.33***  (0.10) |
| Purchasing power in zip code in 10,000€ | --- | --- | 0.42  (0.27) | 0.35  (0.27) | --- |
| % hh with non-German names BY Income in 10,000€ | --- | --- | --- | --- | -0.02  (0.02) |
| Constant | 5.35***  (0.48) | 4.77***  (0.47) | 4.43***  (0.62) | 4.53***  (0.62) | 5.06***  (0.48) |
| Individual and zip-level controls | Yes | Yes | Yes | Yes | Yes |
| Survey year indicators | Yes | Yes | Yes | Yes | Yes |
| Observations | 1,387 | 1, 387 | 1, 387 | 1, 387 | 1, 387 |
| Individuals | 516 | 516 | 516 | 516 | 516 |
| Rho/ICC | .46 | .46 | .45 | .45 | .45 |

Standard errors in parentheses * p<0.1, ** p<0.05, *** p<0.01
