# Supplementary material for: Ethnic diversity, poverty and social trust in Germany: Evidence from a behavioral measure of trust
Source: PLoS One. 2018 Jul 18;13(7):e0199834. doi: 10.1371/journal.pone.0199834 (PMC6051567; doi:10.1371/journal.pone.0199834)
Supplement: S3 Table — (DOCX) [file pone.0199834.s005.docx]

**S3 Table. Ethnic Diversity Indicators at the Kreis-level for all years (2003-2005)**

| **Variable** | **N** | **Mean** | **SD** | **Min** | **Max** | **Description** |
| --- | --- | --- | --- | --- | --- | --- |
| foreigner | 1059 | 7.15 | 4.80 | 1.10 | 26.30 | Share foreigner in district |
| ethno_frac051 | 1059 | 10.49 | 6.33 | 1.23 | 39.21 | Ethnic fractionalization district |
