# Supplementary material for: Ethnic diversity, poverty and social trust in Germany: Evidence from a behavioral measure of trust
Source: PLoS One. 2018 Jul 18;13(7):e0199834. doi: 10.1371/journal.pone.0199834 (PMC6051567; doi:10.1371/journal.pone.0199834)
Supplement: S4 Table — (DOCX) [file pone.0199834.s006.docx]

**S4 Table. Ethnic Diversity Indicators by Survey Year**

| **Survey Year** | **Mean foreigner** | **Mean ethnic fractionalization** |
| --- | --- | --- |
| 2003 | 7.1 | 10.5 |
| 2004 | 7.2 | 10.4 |
| 2005 | 7.2 | 10.5 |
