# Supplementary material for: Ethnic diversity, poverty and social trust in Germany: Evidence from a behavioral measure of trust
Source: PLoS One. 2018 Jul 18;13(7):e0199834. doi: 10.1371/journal.pone.0199834 (PMC6051567; doi:10.1371/journal.pone.0199834)
Supplement: S5 Table — (DOCX) [file pone.0199834.s007.docx]

**S5 Table. Correlations between Ethnic Diversity Indicators at District-Level**

|  | **Foreigner 2003** | **Foreigner 2004** | **Foreigner 2005** |
| --- | --- | --- | --- |
| **ethno_frac051 2002** | 0.83*** |  |  |
| **ethno_frac051 2004** |  | 0.84*** |  |
| **ethno_frac051 2005** |  |  | 0.84*** |
